# Minimally-invasive treatments for benign thyroid nodules: recommendations for information to patients and referring physicians by the Italian Minimally-Invasive Treatments of the Thyroid group

**DOI:** 10.1007/s12020-022-03005-y

**Published:** 2022-03-15

**Authors:** Giovanni Mauri, Stella Bernardi, Andrea Palermo, Roberto Cesareo, the Italian Minimally-Invasive Treatments of the Thyroid group, Enrico Papini, Luigi Solbiati, Daniele Barbaro, Salvatore Monti, Maurilio Deandrea, Laura Fugazzola, Giovanni Gambelunghe, Roberto Negro, Stefano Spiezia, Fulvio Stacul, Luca Maria Sconfienza, Marco Cavallaro, Gaetano Achille, Vito Cantisani, Luca Cozzaglio, Anna Crescenzi, Francesco De Cobelli, Roberto Garberoglio, Gioacchino Giugliano, Luca Persani, Bruno Raggiunti, Ettore Seregni, Dominique Van Doorne, Andrea Frasoldati, Chiara Carzaniga, Celestino Pio Lombardi, Giampaolo Papi, Rinaldo Guglielmi, Franco Orsi, Rosa Cervelli, Chiara Barbieri, Pierpaolo Trimboli, Dario Monzani

**Affiliations:** 1 Dipartimento di Oncologia ed Emato-Oncologia, Università degli Studi di Milano, Milano, Italy; 2 Divisione di Radiologia interventistica, Istituto Europeo di Oncologia, Istituto di Ricovero e Cura a Carattere Scientifico (IRCCS), Milano, Italy; 3 Dipartimento di Scienze Mediche Chirurgiche e della Salute, Università degli Studi di Trieste, Trieste, Italy; 4 UCO Medicina Clinica, Azienda Sanitaria Universitaria Integrata Trieste (ASUGI), Trieste, Italy; 5 Policlinico Universitario Campus Bio-Medico, Roma, Italy; 6 UOS Malattie Metaboliche, Ospedale Santa Maria Goretti, Latina, Italy; 7 Dipartimento di Endocrinologia, Ospedale Regina Apostolorum, Albano Laziale, Italy; 8 Humanitas University, Milano, Italy; 9 UO Endocrinologia ASL Nordovest Toscana, Toscana, Italy; 10 UOC di Endocrinologia, Azienda Ospedaliera Sant’Andrea, Roma, Italy; 11 UO Endocrinologia, Ospedale Mauriziano Torino, Torino, Italy; 12 Istituto Auxologico Italiano IRCCS, Milano, Italy; 13 Università degli Studi di Milano, Milano, Italy; 14 Azienda Ospedaliero-Universitaria di Perugia, Perugia, Italy; 15 UO Endocrinologia, Ospedale “V. Fazzi”, Lecce, Italy; 16 UO Chirurgia Endocrina ed Ecoguidata, Ospedale del Mare, ASL Napoli1, Napoli, Italy; 17 Azienda Sanitaria Universitaria Integrata Trieste (ASUGI), Trieste, Italy; 18 IRCCS Istituto Ortopedico Galeazzi, Milano, Italy; 19 Università degli Studi di Milano, Milano, Italy; 20 IRCCS Oncologico “Giovanni Paolo II”, Unità Operativa ORL, Bari, Italy; 21 Policlinico Umberto I, Università Sapienza, Roma, Italy; 22 Humanitas Clinical and Research Center IRCCS, Rozzano, Italy; 23 UOC Anatomia Patologica, Policlinico Universitario Campus Bio-Medico, Roma, Italy; 24 Ospedale San Raffaele, Università Vita e Salute, Milano, Italy; 25 Ospedale Città della Salute Torino, Dipartimento Universitario di Endocrinologia e Malattie Metaboliche, Torino, Italy; 26 Department of Head and Neck, Istituto Europeo di Oncologia, IRCCS, Milano, Italy; 27 UOC Malattie Endocrine e Diabetologia, ASL Teramo, Teramo, Italy; 28 Fondazione IRCCS Istituto Nazionale dei Tumori, Milano, Italy; 29 AME Associazione Medici Endocrinologi, Udine, Italy; 30 Department of Endocrinology and Metabolism, Arcispedale Santa Maria Nuova IRCCS-ASL, Reggio Emilia, Italy; 31 Division of Endocrine and Metabolic Diseases, San Luca Hospital, Istituto Auxologico Italiano, Milano, Italy; 32 Department of Gastroenterologic, Endocrine-Metabolic and Nephro-Urologic Sciences, Endocrine Surgery Division, Fondazione Policlinico Universitario A. Gemelli IRCCS, Roma, Italy; 33 Unit of Endocrinology, Northern Area, Azienda USL Modena, Modena, Italy; 34 Endocrinology Department, Sant’ Andrea Hospital, Roma, Italy; 35 Divisione di Radiologia interventistica, Istituto Europeo di Oncologia, Istituto di Ricovero e Cura a Carattere Scientifico IRCCS, Milano, Italy; 36 Division of Interventional Radiology, Azienda Ospedaliero Universitaria Pisana, Pisa, Italy; 37 European Patients’ Academy on Therapeutic Innovation (EUPATI) Trained Patient, Utrecht, The Netherlands; 38 Servizio di Endocrinologia e Diabetologia, Ospedale Regionale di Lugano, Ospedale Regionale di Lugano, Ente Ospedaliero Cantonale (EOC), Lugano, Switzerland; 39 Facoltà di Scienze Biomediche, Università della Svizzera Italiana (USI), Lugano, Switzerland; 40 Dipartimento di Oncologia ed Emato-Oncologia, Università degli Studi di Milano, Milano, Italy; 41 Divisione di Psiconcologia, Istituto Europeo di Oncologia, IRCCS, Milano, Italy

**Keywords:** Thyroid nodule, Patient, Radiofrequency, Laser, Ethanol, Microwave

## Abstract

**Purpose:**

In this paper, the members of the Italian Working Group on Minimally-Invasive Treatments of the Thyroid (MITT group) aim to summarize the most relevant information that could be of help to referring physicians and that should be provided to patients when considering the use of MITT for the treatment of benign thyroid nodules.

**Methods:**

An interdisciplinary board of physicians with specific expertise in the management of thyroid nodules was appointed by the Italian MITT Group. A systematic literature search was performed, and an evidence-based approach was used, including also the knowledge and the practical experience of the panelists to develop the paper.

**Results:**

The paper provides a list of questions that are frequently asked by patients to operators performing MITT, each with a brief and detailed answer and more relevant literature references to be consulted.

**Conclusions:**

This paper summarizes the most relevant information to be provided to patients and general practitioners/referring physicians about the use of MITT for the treatment of benign thyroid nodules.

## Introduction

Thyroid nodules are a very common occurrence in the general population. In most cases, thyroid nodules are benign, however, even if benign, they can grow and determine compressive symptoms or cosmetic concerns, thus requiring a treatment. Surgical removal of half or the whole thyroid gland represents the standard treatment for symptomatic benign thyroid nodules [1–3].

In the last years, minimally-invasive treatments for the thyroid (MITT), including ethanol ablation (EA) and thermal ablation (TA), have gained an increasingly promising role in the management of symptomatic benign thyroid nodules and nowadays they are included as therapeutic alternatives to surgical excision in several guidelines [4–8]. However, despite the increasing evidence of safety and efficacy of MITT for benign thyroid nodules, there is still a large variation in the application of the different available techniques among different countries [9], and not uniform knowledge among patients and referring physicians on MITT. In order to overcome this issue, in February 2018, during a meeting involving different specialists with specific expertise in the use of MITT, the Italian MITT Group was founded, aiming at improving research and diffusion of MITT [10].

This paper has been ideated by the Italian MITT group members to summarize the most relevant information that patients and general practitioners/referring physicians should know about the use of MITT for the treatment of benign thyroid nodules. This paper is in no way intended to replace a detailed patient information and consent form.

## Materials and methods

For this consensus, an interdisciplinary board of physicians with specific expertise in the management of thyroid nodules was appointed by the Italian MITT Group for their experience in clinical practice and research on the topic and willingness to participate. The panel included radiologists, endocrinologists, nuclear medicine physicians, pathologists, surgeons and an European Patients’ Academy on Therapeutic Innovation (EUPATI) trained patient (CB). Four authors (GM, SB, AP, RC) conceived the initial draft of the paper, which was then disseminated to all the participants for comments and consideration. After discussion, a final draft was elaborated and sent for final approval to all the participants. In order to make the text easier to read also for patients, it has been organized in a list of frequently asked questions, with their respective answers.

## Frequently asked questions

### What are the “minimally-invasive treatments of the thyroid”?

The “minimally-invasive treatments of the thyroid” or MITT are a group of interventions that may be used in the treatment of benign thyroid nodules, and that are characterized by a minimally-invasive approach.

MITT are considered as minimally-invasive because they do not require any general anesthesia, or surgical incision, or the removal of healthy thyroid tissue, thus allowing for a minimal impact on patients while providing an effective treatment of the disease. Given that they are provided under the guidance of ultrasound, MITT are also called “ultrasound-guided treatments”.

MITT include both chemical, such as EA, and thermal ablations (TA), the latter comprising four different techniques, namely radiofrequency ablation (RFA), laser ablation (LA), microwave ablation (MWA), and high-intensity focused ultrasound (HIFU). These techniques differ in terms of mode of action, type of energy applied, and the devices used for their application. In case of EA, RFA, LA and MWA a small probe (21–17G) is inserted through the skin into the thyroid nodule to be treated, whereby ethanol or thermal energy are delivered directly into the nodule, to determine cellular death. With HIFU, a device is applied to the neck of the patient, and energy focused on the nodule without even the need of skin incision.

EA, RFA and LA are the most widely used techniques in the world, and -among thermal ablations-RFA and LA have the larger scientific evidence [11–15]. MWA is currently mainly used in Asia. HIFU is currently available in very few Centers in the world, the technology being still in its infancy.

### When can a thyroid nodule be treated with MITT?

The use of MITT is indicated for the treatment of benign thyroid nodules that are symptomatic. It may be considered in benign nodules that tend to grow. Also, MITT, particularly TA, can be used in the treatment of autonomously functioning thyroid nodules in selected cases.

MITT are indicated when thyroid nodules become symptomatic and cause local discomfort or cosmetic concerns. They can be taken into account also in nodules that tend to grow, provided that they are cytologically benign.

Thyroid cysts do not need additional exams other than an US before their treatment. In all the other cases, before undergoing any of these procedures, it is important to ascertain what is the nature of the nodule to treat and verify that it is cytologically benign. This is usually performed by fine needle aspiration cytology (FNAC). In case of nodules with US features indicating a low risk of malignancy or autonomously functioning thyroid nodules (AFTN), a single FNAC is considered sufficient before their treatment, while it should be repeated in all the other cases to confirm that the nodule is cytologically benign [4, 6]. MITT are not indicated to treat nodules that are cytologically suspicious for malignancy or malignant—except for few selected cases of patients unsuitable for surgery or patients with microcarcinomas [16]. MITT can be used to treat autonomously functioning nodules, and -in this case- it is particularly effective for small (<10 ml) nodules.

### How to choose a specific MITT technique over another?

The choice of the technique to use is based on nodule characteristics, local technique availability and physician’s expertise. In general, EA is indicated for cystic or predominantly cystic nodules, while the other techniques, particularly RFA and LA, are indicated for predominantly solid or solid nodules.

The specific technique to use depends on nodule US structure. Thyroid nodules can be classified as solid (<10% of fluid component), predominantly solid (11–50% of fluid component), predominantly cystic (51–90% of fluid component), or cystic (>90% of fluid component) [13, 17]. Spongiform nodules are defined as nodules containing multiple small cysts <5 mm interspersed within solid tissue for nearly all the volume. EA is the preferred technique to use in case of cystic or predominantly cystic thyroid nodules, because it has fewer side effects and lower costs as compared to the other techniques [8]. The other techniques, particularly RFA, LA, and MWA are indicated for the treatment of spongiform, predominantly solid, or solid nodules [18, 19].

In case of AFTN, radioactive iodine remains the first choice treatment modality [1, 3]. However, TA can be performed, also in combination with radioiodine (RAI) ablation, and good results on compressive symptoms and thyroid function normalization have been reported in the literature [20].

### How is ethanol ablation (EA) of a thyroid cystic nodule performed?

EA consists of the injection of ethanol into a cystic (or a predominantly cystic) nodule, once its liquid content has been aspirated.

EA is an outpatient procedure. The patient lies on the operating table with the neck extended, and under ultrasound guidance local anesthesia is performed. Then, the thyroid cyst is punctured under continuous ultrasound visualization, and the cystic fluid is aspirated to the maximum extent possible. Once the fluid component has been aspirated, ethanol is slowly injected into the cystic cavity. Usually, ethanol is injected in an amount corresponding to ~50% of the aspirated liquid volume. After EA, a small plaster is applied to the neck, and the patient remains under observation for about 30 min before returning to activity [21, 22].

### How is a thermal ablation (TA) of a benign thyroid nodule performed?

TA consists of the ablation of a solid (or predominantly solid) nodule with the application of thermal energy to the nodule.

With LA, RFA and MWA thermal energy is delivered through small devices that are inserted under ultrasound guidance into the thyroid nodule. By contrast, in case of HIFU, ultrasound waves at high energy are focused on the thyroid nodule to treat, with no need to insert any probe/needle into it. All these procedures are performed in an outpatient setting, without the use of general anesthesia. Local anesthesia is generally used for TA, although some authors reported the use of LA or HIFU without local anesthesia. Sometimes, mild sedation can be performed. During the procedure, the patient should lay on the operatory table with the neck extended. Under ultrasound guidance the operator injects a small amount of local anesthetic around the thyroid capsule and into the surrounding soft tissues of the neck. Then, according to the technique applied, one or more small devices are inserted under ultrasound guidance into the thyroid nodules, or the HIFU device is applied to the patient neck, and the ablation is started. With RFA and MWA a single device is inserted into the nodule, and moved several times to achieve ablation of the whole nodule (moving-shot technique), while with LA up to 4 very thin laser fibers are inserted simultaneously into the deeper part of the nodule and then withdrawn in order to achieve the complete ablation of the nodule (pull-back technique). With HIFU, no device is inserted into the thyroid nodule, as the energy is delivered directly through the skin. According to the volume of the nodule and the technique used, the ablation can last from few minutes to up to 30 minutes approximately. During the procedure, the patient can speak to the operator, and the ablation can be stopped immediately in case of discomfort or pain. At the end of the procedure, a plaster and an ice pack are applied to the neck of the patient. The patient should remain under observation for a couple of hours before being discharged. In general, patients can return to their usual activities the day after the intervention. In case of very large nodules, or particularly fragile patients, a couple of days of rest is suggested [15, 23].

### How should a patient be prepared for the procedure?

The only special precautions that must be taken before the procedure concern patients with voice changes, previous neck surgery, patients on oral anticoagulants or antiplatelet drugs, and patients with pacemakers or implantable-cardioverter defibrillators (in case RFA is performed with monopolar electrodes).

Patients presenting with voice changes should be evaluated further to exclude the presence of a vocal cord palsy prior to the procedure, as if it is contralateral to the nodule of interest, it is a relative or absolute contraindication to TA (depending on its topographic location). Particularly, before the procedure, thyroid and vocal cord function, comorbidities, and contraindications to treatment should be evaluated; laryngoscopy is recommended in patients with hoarseness, previous neck surgery, or with nodules close to critical structures.

Although MITT carry a low risk of major bleeding, oral anticoagulants or antiplatelet drugs can lead to prolonged bleeding or bruising. Before the procedure, it is recommended withholding oral anticoagulants. Patients with a high-intermediate thromboembolic risk require bridging warfarin with low molecular weight heparin, while patients with low thromboembolic risk do not. Given the low risk of major bleeding, anticoagulants can be resumed at maintenance dose on the evening after the procedure (in association with low molecular weight heparin in case of high-intermediate thromboembolic risk) [24]. Low-dose aspirin should not be discontinued before the procedure given the low bleeding risk of MITT. Patients on dual antiplatelet therapy should have the procedure postponed until the duration of the dual therapy is finished [25]. New oral anticoagulants, which include dabigatran, rivaroxaban, apixaban, and edoxaban, should be hold for 24–36 h and can be safely resumed 24 h after the procedure.

RFA performed with monopolar electrodes may cause electromagnetic interferences, as the heat at the tip of the electrode is produced by an alternating electric field, which might interfere with cardiac pacemakers (PM) or implantable-cardioverter defibrillators (ICD) functions. In patients with a cardiac activity that is not PM-dependent, special precautions are not needed [26]. In patients with a cardiac activity that is PM-dependent, the operator should place a magnet over the device and peripheral pulse should be monitored during the procedure. The magnet placed over the cardiac region is considered to be safer than any PM reprogramming, as it allows an immediate restoration of the PM functions after the procedure. Anyway, bipolar RFA electrode can be used to overcome these issues. Only patients with ICD should have their tachyarrhythmia treatment algorithms programmed off before the procedure. In this case, patients should be monitored during the procedure, and straight after RFA, the ICD should be programmed back to its original settings.

### What may a patient expect after the procedure?

Patients may briefly experience mild pain in the days following the treatment; which usually extends as far as the jaw and can be relieved by oral painkillers.

Immediately after the procedure, it is recommended to apply an ice pack on the neck with mild pressure, and to keep the patient under observation for a couple of hours. Generally, there is no need of medication after the procedure. A minority of patients (~10%) may experience neck pain [27, 28] with sensation of local heat sometimes radiating to the jaw, ipsilateral dental arch, mandibular angle, or shoulder. In case of persistent neck pain, oral painkillers such as paracetamol are usually sufficient to relieve patients’ symptoms [27]. In addition, it is recommended avoiding strenuous physical exercise, as well as overextending the throat in the first few days after MITT in order to support the healing process. Otherwise, there are no restrictions on patients’ usual daily routine.

### What happens to the nodule after MITT?

After MITT (EA or TA) the nodule starts to reduce in size progressively.

With EA, due to the aspiration of the fluid component, the nodule is immediately reduced in size after the treatment, with an immediate improvement of patients’ symptoms. In the following time, the nodule continues to slightly reduce its volume, and generally it achieves a volume reduction of 60 to 80% at 6 months from treatment [8, 21, 22]. The technique efficacy of EA, which is defined as a volume reduction of at least 50% as compared to the initial nodule volume, is generally achieved in as much as 90% of cases. In case of an insufficient volume reduction, or persistence of compressive symptoms, EA can be easily repeated to achieve the desired result.

By contrast to EA, straight after TA the nodule might appear even slightly enlarged due to the oedema occurring after the treatment, and patients might not experience an immediate benefit. It is ~1 week after the procedure, when the nodule starts to shrink, that patients start feeling a relief from their symptoms. Then, the nodule continues to decrease in size, with a parallel symptom improvement, up to its maximum volume reduction, which is usually achieved between 6 and 24 months after the treatment. Final volume reduction usually ranges from 60–80%, and this result is generally maintained over time [12, 29]. The technique efficacy of TA is generally achieved in as much as 90% of cases. After treatment, a short period follow-up (after 1 to 3 months from the procedure) with neck US, thyroid test function and clinical visit is generally recommended. Afterwards, patients are generally followed at 6 and 12 months, and every year onward.

### Which side effects and complications might occur after MITT?

The overall likelihood of modest or severe complications is low, and -when occurring- complications are in the vast majority of cases of minor entity. The most common complications and side effects are bruising and mild pain in the neck. The risk of scarring is minimal. In exceptional cases, there may be temporary hoarseness or nodule rupture and infections.

A low rate of side effects and complications has been reported in the literature [27, 30, 31]. Side effects (7–30%) include: pain, bruising, fever, nodule swelling, cough, and vasovagal reactions. Complications (1–3.5%) may be divided into immediate and delayed. Immediate complications include: hoarsenes/voice changes, and skin burns. Delayed complications include: bruising, nodule pseudocystic transformation (also known as nodule rupture) with or without fasciitis, and thyroid dysfunction. Hoarseness/voice changes occur in 0.8–2.4% of patients. They can be due to the thermal injury to the laryngeal nerve or to its compression caused by perinodular edema and generally they resolve spontaneously with the administration of corticosteroids. Also cold solution of 5% dextrose may be injected close to the nerve route as a rescue maneuver [32]. The risk of skin burn is extremely low [27, 30, 33]. In order to avoid it, it can be useful to inject cold fluid in the subcutaneous layers in order to create a cushion that will raise the skin and increase the distance from the nodule [34]. Bruising occurs in 0.8–2.5% of patients, and generally it appears within a couple of hours-days after the procedure, disappearing in a few days-weeks [27, 33]. Pseudocystic transformations/nodule rupture occur in 0.3–4.9% of patients. They manifest as a painful sudden swelling a few days after the procedure. In case of fever and persistence of symptoms, patients should be treated with antibiotics and analgesics [30, 33, 35]. A few isolated cases of transient or immunogenic forms of hyperthyroidism have been reported. In the case of hypothyroidism it is unclear whether this represents part of the natural history of an underlying thyroid pathology or if it is a consequence of MITT. Nevertheless, thyroid function should be monitored at the time of the first follow-up or whenever clinical suspicion of dysfunction arises [33, 36].

### Can the nodule regrow after the procedure?

After MITT, the treated nodule usually shrinks and changes US features, but a small “remnant” nodule remains. Over time, some nodules might regrow. Regrowth is often asymptomatic and does not necessarily require further treatments.

MITT are very effective in reducing thyroid nodule volume. Nodule shrinkage is associated with change of its US features, as it becomes hypoechoic and avascular. A small “remnant” nodule remains after the procedure. Based on the literature, regrowth (which should be defined as a > 50% increase as compared to the smallest recorded volume) occured in as much as one third of patients in a retrospective 5-year analysis [13]. Regrowth is often asymptomatic and does not require further treatments. In case of rapid nodule regrowth, clinicians might consider US-guided FNAC.

### It is possible to perform more than one treatment?

Thyroid nodules can be treated more than once with MITT.

Thyroid nodules can be treated with MITT multiple times in order to achieve the desired clinical outcome. Baseline volume may be used to predict how many treatments are needed to achieve a satisfactory nodule reduction. In particular, it has been suggested that one session is enough for small nodules, while 2 or 3 sessions may be required for medium or large size nodules [36, 37]. In case of predominantly cystic nodules treated with EA, it is possible that more than one treatment will be necessary over time. However, the decision of performing more than one MITT session should be based on patient’s complaints (symptoms and cosmetic concerns) rather than the amount of nodule volume reduction, and it should be limited to patients with unresolved clinical problems.

### Will the procedure impact on future thyroid surgery?

The surgical removal of thyroid nodules appears to be minimally impacted by MITT

After MITT, radioactive iodine and—most importantly—surgery remain both feasible. In one publication on the topic, it has been shown that RFA did not affect subsequent thyroid surgery and/or histological diagnosis [38]. To ensure that surgical removal of thyroid nodules is not impacted by RFA (or MWA), the active tip of the needle should be kept within the nodule throughout the procedure, allowing the operator to undertreat the areas adjacent to the capsule [38], and preventing possible complications. However, some surgeons anecdotally reported a higher difficulty to mobilize the anterolateral side of the thyroid lobe, while they had no problems with the posterior and medial sides (because here the capsule is less likely involved by the intervention).

### What are the pros and cons of MITT in comparison to surgery?

MITT are outpatient procedures with good efficacy, minimal recovery time, and few adverse events. They do not require levothyroxine replacement therapy. On the other hand, a small remnant nodule may remain, MITT do not allow for final pathology, and subsequent treatments may be required.

Standard thyroid surgery is invasive, requires general anesthesia, and has some drawbacks. The duration of a procedure is 2 to 3 h, it requires a hospital stay of 1 or 2 days and the recovery time is usually 1 to 2 weeks. Surgery leads to complete removal of the thyroid nodule, which allows for final pathology, and usually there is no need to repeat the procedure. On the other hand, it leaves a neck scar, it often requires the introduction of levothyroxine replacement therapy, and it carries a higher risk of permanent voice damage, bleeding complications and postoperative pain and -in the case of thyroidectomy- also of hypoparathyroidism [36]. MITT are a therapeutic alternative for the treatment of thyroid nodules that can be performed in an outpatient setting with minimal recovery time and few adverse events. MITT are less invasive, they do not require general anesthesia and have a lower risk of complications. The duration of a procedure is 30 minutes to 1 hour, and the recovery time typically is 1 to 2 days. MITT do not leave scars, do not require replacement therapy, and the risk of permanent voice damage is very low. However, a small nodule remains, requiring a follow-up over time and in some cases to repeat the procedure. In addition, MITT do not allow for final pathology as surgery does, with the risk of missing indolent low-risk cancers.

### What are the pros and cons of MITT in comparison to radioactive iodine for the treatment of AFTN?

Radioactive iodine therapy is the gold standard for the treatment of autonomously functioning thyroid nodules. MITT can be used in a few selected cases instead of RAI.

Radioactive iodine therapy is one of the first line treatments for AFTN [3, 39]. RAI therapy with I-131 is given to destroy overactive thyroid tissue and treat hyperthyroidism. Patients are asked to follow some radiation precautions after treatment in order to limit radiation exposure to others, particularly to pregnant women and young children. RAI cannot be used in pregnant or breastfeeding women. RAI may take up to several months to have its effect and in some cases the final result is hypothyroidism, which requires hormone replacement therapy. MITT can be used for the treatment of small AFTN [1, 40]. Its advantages include a rapid reduction of nodule volume [41], and no need of hormonal replacement therapy. Also, MITT can be applied in pregnant and breastfeeding women. Its disadvantages include primarily the lower efficacy in thyroid function normalization as compared to RAI [42], as well as a very low risk of adverse events (voice change). As a rule of thumb, MITT should be used as a treatment for AFTN only in selected cases, preferably in small AFTN when RAI is contraindicated (pregnant and breastfeeding women) or refused by patient, or in women with an immediate desire to conceive.

## Abbreviations

MITT: Minimally invasive treatments for the thyroid
EA: Ethanol ablation
TA: Thermal ablation
RFA: Radiofrequency ablation
FNAC: Fine needle aspiration citology
HIFU: High Intensity focused ultrasound
LA: Laser ablation
MWA: Microwave ablation
AFTN: Autonomously functioning thyroid nodules
US: Ultrasound
PM: Pacemaker
ICD: Implantable cardioverter
RAI: Radioactive iodine
